# Editorial: Liver diseases – from pathophysiology to new treatment options

**DOI:** 10.3389/fimmu.2026.2020350

**Published:** 2026-10-20

**Authors:** Theodoros Androutsakos, Narjes Nasiri-Ansari, Margarita Papatheodoridi, Dimitrios Karagiannakis

**Affiliations:** 1 Department of Pathophysiology, Medical School, National and Kapodistrian University of Athens, Athens, Greece; 2 Division of Cardiology, Pulmonology, and Vascular Medicine, University Hospital and Medical Faculty, Heinrich Heine University, Düsseldorf, Germany; 3 Academic Department of Gastroenterology, Medical School of National and Kapodistrian University of Athens, Athens, Greece; 4 Fourth Department of Internal Medicine, “Attikon” University Hospital, School of Medicine, National and Kapodistrian University of Athens, Athens, Greece

**Keywords:** autoimmune liver diseases, hepatocellular carcinoma, liver cirrhosis, liver disease, steatotic liver disease (SLD), viral hepatitis

## 1 Introduction

Liver diseases are a major global health challenge, spanning viral, autoimmune, cholestatic, metabolic, and alcohol-associated diseases, with progression to cirrhosis, decompensation, and hepatocellular carcinoma (HCC). Advances in molecular biology, genetics, immunology, and systems biology are refining our understanding of disease progression and informing new diagnostic, prognostic, and therapeutic strategies.

This Frontiers in Immunology Research Topic brought together basic, translational, and clinical studies focused on inflammation, metabolic and immune dysregulation, fibrogenesis, biomarkers, non-invasive approaches, and emerging therapies. Collectively, these studies highlight both shared biological pathways and disease-specific mechanisms relevant to precision diagnosis and treatment.

## 2 Viral hepatitis

Viral hepatitis remains a major cause of liver-related morbidity and mortality, with more than 300 million people worldwide living with chronic hepatitis B (HBV) or hepatitis C (HCV) infection (1–4). Despite major advances in HBV vaccination, long-term antiviral suppression, and highly effective curative therapy for HCV, substantial gaps in diagnosis, linkage to care, and treatment coverage persist globally.

### 2.1 HBV

HBV infection follows a complex, dynamic natural history characterized by overlapping clinical phases shaped by host immune response, viral replication, and age at infection (5).

Chen et al. reviewed mechanisms underlying innate immune recognition and HBV evasion strategies. They summarized how pattern-recognition receptors sense HBV-derived DNA and RNA, including pregenomic RNA, as well as viral proteins, with particular emphasis on Toll-like receptors and retinoic acid-inducible gene I (RIG-I)-like pathways. The authors further discussed how HBV interferes with these sensing mechanisms to establish persistent infection. By delineating the interplay between viral components and host innate immunity, this review highlights pattern-recognition receptor pathways as potential targets for future immunomodulatory approaches.

Immune dysfunction, namely immune paralysis, characterized by impaired innate immune responses, compromised antimicrobial defense, and increased susceptibility to secondary infections, was the focus of Liu et al.’s study of patients with HBV-related acute-on-chronic liver failure (HBV-ACLF). The authors identified Methyl-CpG-binding domain protein 2 (MBD2) as significantly upregulated in HBV-ACLF monocytes, with expression levels correlating with disease severity, systemic inflammation, and clinical outcome. Mechanistic studies showed that loss of MBD2 increased chromatin accessibility and transcription of genes involved in antigen presentation and pro-inflammatory responses, including pathways related to major histocompatibility complex (MHC) class II synthesis. These findings suggest that MBD2 epigenetically represses HLA-DR expression in monocytes, leading to impaired antigen presentation and immune paralysis. The authors proposed MBD2 as a potential biomarker and therapeutic target for restoring immune competence in ACLF.

Continuing with HBV-oriented immune responses, Li et al. contributed a manuscript aimed at identifying characteristic hub genes in the immune-active phase of chronic hepatitis B, defined by positive serum HBsAg, elevated alanine transaminase (ALT) levels, and detectable HBV DNA. By integrating transcriptomic analyses, weighted gene co-expression network analysis, and machine-learning approaches, the authors identified four candidate hub genes—CCR5, IL10RA, KCNA3, and SLC24A4—associated with the immune-active phase. Among these, SLC24A4 emerged as a particularly relevant immune-regulatory candidate. In silico knockout analyses suggested that SLC24A4 may act as a negative regulator of monocyte activation, highlighting SLC24A4 as a potential biomarker and therapeutic target for immune-modulatory strategies in chronic HBV infection.

Progressive development of liver fibrosis, which can eventually lead to cirrhosis, hepatic decompensation, and hepatocellular carcinoma, is a key determinant of long-term prognosis in patients with chronic hepatitis B (6).

To unravel this process, Wu et al. investigated the role of Janus kinase-signal transducer and activator of transcription (JAK–STAT) signaling in HBV-related fibrogenic progression. Using an integrated bioinformatics approach, the authors identified CCND1 and IL7R as key JAK–STAT pathway-associated genes in HBV-related fibrosis. Functional analyses further indicated that CCND1 and IL7R are involved in immune regulatory and profibrotic pathways and are closely associated with M1 macrophages and other immune cell subsets, thereby identifying CCND1 and IL7R as potential biomarkers and molecular targets in HBV-associated liver fibrosis.

Current treatment of HBV infection relies primarily on potent nucleos(t)ide analogs and, in selected patients, pegylated interferon-α (Peg-IFN-α); however, achieving functional cure, defined as sustained HBsAg loss, remains uncommon, underscoring the need for reliable predictors of treatment response and strategies that optimize efficacy while minimizing treatment-related toxicity (7, 8).

Two contributions to this Research Topic specifically addressed these issues in relation to Peg-IFN-α. Pang et al. studied 91 HBV-infected individuals to test whether early on-treatment expression of ectonucleotide pyrophosphatase/phosphodiesterase 1 (ENPP1) and interferon-induced protein with tetratricopeptide repeats 2 (IFIT2) in peripheral blood mononuclear cells could serve as biomarkers to predict hepatitis B surface antigen (HBsAg) clearance and therapeutic responses. Both ENPP1 and IFIT2 mRNA levels increased in patients with virological and serological responses; their expression levels at weeks 12 and 24 positively correlated with HBsAg reduction, and their early induction at week 12 was associated with subsequent HBsAg decline. Furthermore, combining ENPP1 and IFIT2 improved predictive performance, supporting their potential utility as early biomarkers of Peg-IFN-α response.

Along these lines, Zhu et al. reviewed the mechanisms and management of Peg-IFN-α toxicity in chronic HBV infection. The authors describe the most common and serious adverse effects, explain the pathophysiological mechanisms behind each, and, in the final section, discuss predictive biomarkers, risk prediction, and personalized management. The review therefore provides a clinically oriented framework for anticipating, monitoring, and managing Peg-IFN-α-associated toxicity, with particular emphasis on individualized risk assessment.

Jin et al. provided a comprehensive bibliometric overview of the evolving field of immunotherapy for chronic HBV infection. They discussed the growing interest in immune-based approaches to an HBV cure. China and the United States emerged as the leading contributors. At the same time, keyword and citation analyses revealed growing interest in the persistence of viral reservoirs, profound immune dysfunction and exhaustion, and mechanisms linking chronic HBV infection to hepatocellular carcinoma. Notably, the literature landscape indicates a clear shift toward combination strategies that integrate potent viral suppression with immune modulation.

### 2.2 HCV

Despite the highly effective direct-acting antiviral (DAA) treatment, HCV remains a major cause of global morbidity and mortality (2). Although DAA treatment has led to successful HCV eradication in more than 95% of patients, regardless of comorbidities (9), the presence of cirrhosis and/or portal hypertension poses a significant clinical challenge.

In their published study, Martin-Escolano et al. investigated the immunological determinants of persistent portal hypertension after HCV cure in patients with advanced cirrhosis, with particular emphasis on the impact of HIV coinfection. In this prospective study of 41 patients, baseline immune profiles were associated with changes in the hepatic venous pressure gradient over 48 weeks. In patients without HIV, impaired portal pressure regression was linked to systemic inflammation and broad CD4+ T-cell activation. In contrast, HIV-coinfected patients exhibited a distinct profile marked by endothelial dysfunction and activation of effector-memory and TemRA CD4+ T cells. These findings suggest that HIV reshapes the immunological mechanisms governing hepatic recovery after HCV eradication and may help identify pathways that contribute to residual portal hypertension despite virological cure.

## 3 Autoimmune liver diseases

Autoimmune liver diseases (AILDs) encompass a heterogeneous group of chronic inflammatory disorders, including autoimmune hepatitis (AIH), primary biliary cholangitis (PBC), and primary sclerosing cholangitis (PSC), with a steadily rising worldwide prevalence (10). Despite substantial advances in understanding their immunopathogenesis, the mechanisms that drive disease initiation, progression, and phenotypic heterogeneity remain incompletely understood (11–13).

Zhang’s review focused on the complex pathogenesis of these diseases, namely the AIH-PBC and AIH-PSC variants. The author described distinct immune architectures characterized by T-cell dysfunction, pro-inflammatory macrophage niches, B-cell activation, tertiary lymphoid structures, and senescent cholangiocytes. As a result, persistent crosstalk among adaptive immune cells, the biliary epithelium, bile acid signaling, and inflammatory pathways may sustain liver injury despite conventional immunosuppression. The review also highlights emerging precision approaches, including JAK inhibition and B-cell–depleting therapies, for difficult-to-treat disease.

To better understand AILD pathogenesis, Wang et al. examined the significance of ductopenia in PBC. In a study of 518 patients with biopsy-proven PBC, ductopenia was common, affecting 56.76% of patients, including 24.83% with early-stage disease. In multivariable logistic regression, serum ANA ≥1:1000, elevated gamma-glutamyl transferase and total bilirubin, and low serum albumin emerged as biomarkers for ductopenia in patients with early-stage PBC. During follow-up, patients with ductopenia had a higher prevalence of liver-related adverse events, underscoring the significance of ductopenia in disease progression.

PBC has recently attracted significant interest due to the rapid expansion of second-line treatments, including the two approved peroxisome proliferator-activated receptor (PPAR) agonists, elafibranor and seladelpar, as well as several additional agents in clinical development (14).

Against the rapidly evolving therapeutic landscape, Angelara et al. reviewed current and emerging treatment options for PBC. The authors emphasize that a substantial proportion of patients fail to achieve an adequate biochemical response to first-line ursodeoxycholic acid and discuss the efficacy, safety, and positioning of recently introduced PPAR agonists. They also review emerging agents targeting FXR, FGF19/FGFR4, NOX1/4, immune pathways, and bile acid transport, illustrating the ongoing shift toward more individualized, mechanism-based treatment strategies.

Immunoglobulin G4-related disease (IgG4-RD) is another autoimmune disease that commonly affects the biliary tract but usually spares the liver (15). Wang et al. reported a rare case of seronegative, IgG4-related hepatic inflammatory pseudotumor (IgG4-HIPT) in a 60-year-old man with multifocal hepatic lesions, mild inflammatory symptoms, and normal serum IgG4 levels. Histopathological analysis showed dense IgG4-positive lymphoplasmacytic infiltration, storiform fibrosis, and obliterative phlebitis, establishing the diagnosis of IgG4-HIPT, which responded well to corticosteroids. The authors also discuss the immunopathogenesis, significance, and differential diagnosis of this rare entity. Beyond its rarity, the case highlights the potential dissociation between circulating biomarkers and tissue-level immune activity and underscores the importance of histological assessment in the differential diagnosis of atypical hepatic lesions.

## 4 Metabolic dysfunction-associated steatotic liver disease

Metabolic dysfunction and alcohol exposure are increasingly important drivers of chronic liver disease and share key mechanisms with other forms of liver injury, such as oxidative stress, inflammation, immune dysregulation, and hepatocellular damage (16, 17).

MASLD is a leading cause of chronic liver disease, encompassing steatosis, steatohepatitis, fibrosis, cirrhosis, and HCC, and is closely associated with cardiovascular and metabolic disease (17). Contributions to this Research Topic span clinical and metabolic markers, immune mechanisms, and experimental therapies.

Al-Khinji et al. examined hematological abnormalities in patients with NAFLD, particularly those with clinically diagnosed non-alcoholic steatohepatitis (NASH), in a large case-control cohort from Qatar. Hematological abnormalities, particularly thrombocytopenia, were more prevalent among patients with NASH, and higher fibrosis-4 (FIB-4) index values were independently associated with anemia and abnormal platelet counts. The progressive increase in platelet abnormalities across higher FIB-4 risk categories further supports the potential value of readily available hematological parameters for identifying patients at increased risk of advanced fibrosis.

Moving from conventional clinical biomarkers toward molecular characterization, Huang et al. investigated the evolving hepatic immune landscape across the NAFLD spectrum using single-cell transcriptomic datasets. Plasmacytoid dendritic cells (pDCs) exhibited prominent stage-dependent changes, with a subset expressing granzyme B (GZMB) and tropomyosin 2 (TPM2) enriched in NASH. A pDC-derived diagnostic model subsequently discriminated NASH across validation cohorts. This work shows that characterizing immune-cell remodeling may complement conventional clinical markers and help develop molecular biomarkers for steatohepatitis.

Chen et al. further highlighted the close intersection among metabolic, cardiovascular, and hepatic diseases by evaluating the triglyceride-glucose (TyG) index and the triglyceride-glucose-body mass index (TyG-BMI) in patients undergoing percutaneous coronary intervention. Both indices were independently associated with NAFLD and correlated with higher NAFLD fibrosis scores. These findings suggest that simple metabolic indices reflecting insulin resistance and adiposity may help identify cardiovascular patients who warrant further evaluation for steatotic liver disease and fibrosis.

The Research Topic also expanded from disease characterization to potential therapeutic approaches. Xu et al. investigated Tao-Hong-Si-Wu-Tang (THSWT) in an experimental model of diet-induced hepatic steatosis. They demonstrated improvements in steatosis, aminotransferases, and fibrosis, along with modulation of lipid metabolism, oxidative stress, inflammatory signaling, apoptosis, and pyroptosis. Although these findings require clinical validation, they further underscore the interconnected roles of metabolic dysfunction, innate immunity, and regulated cell death in steatotic liver injury.

Ye et al. broadened this therapeutic perspective by reviewing the potential complementary effects of exercise and natural products in NAFLD/MASLD. Natural compounds such as resveratrol, curcumin, and silymarin may influence lipid metabolism, oxidative stress, and inflammatory pathways. In contrast, aerobic and resistance exercise may improve insulin sensitivity, reduce intrahepatic fat, and enhance metabolic function. The authors also highlight microbiota modulation and the gut–liver axis as potential mechanisms underlying these effects. However, clinicians still need stronger clinical evidence before routinely recommending specific combinations, doses, or exercise regimens.

## 5 Alcohol-associated liver disease

Alcohol remains a major cause of chronic hepatocellular injury (18). However, MASLD and ALD have distinct initiating factors; both share mechanisms of oxidative stress, inflammation, immune dysregulation, metabolic alterations, and progressive hepatocellular damage (19).

Zhou et al. used an integrated multi-omics strategy to distinguish potential causal mediators of ALD from merely associated biomarkers. Mendelian randomization, followed by genetic, single-cell transcriptomic, and experimental analyses, identified four compelling proteins: triggering receptor expressed on myeloid cells-like 2 (TREML2) and matrix metallopeptidase 12 (MMP12), which were associated with increased ALD risk, and phospholipase A2 receptor 1 (PLA2R1) and MYC-associated factor X (MAX), which appeared protective. The localization of risk-associated proteins predominantly to hepatic myeloid cells further supports the importance of inflammation and matrix remodeling in ALD and identifies potential targets for future therapeutic investigation.

## 6 Liver fibrosis

Chronic liver diseases of diverse etiologies converge on inflammation, hepatic stellate cell activation, and extracellular matrix accumulation, making fibrosis a central process that links disease-specific injury to advanced chronic liver disease (20).

Lu et al. reviewed how multi-omics technologies are reshaping our understanding of hepatic fibrosis and cirrhosis across etiologies, including viral hepatitis, ALD, and MASLD. Integrating transcriptomic, proteomic, and metabolomic data highlights common mechanisms, such as hepatic stellate cell activation, extracellular matrix deposition, immune dysregulation, oxidative stress, and metabolic alterations. These approaches may uncover molecular pathways, biomarkers, and therapeutic targets that conventional methods do not readily identify. At the same time, cross-species comparisons underscore both the translational value and the limitations of experimental models.

Persistent fibrogenesis culminates in cirrhosis, marked by architectural distortion and major vascular, metabolic, and immune dysfunction (21). Contributions to this Research Topic address both the molecular diagnosis of rare inherited diseases and the changing epidemiology of cirrhosis at the population level.

Nuzhnaya et al. broadened the spectrum of FOCAD-associated infantile liver disease by identifying a novel homozygous splice-site variant in a child with early-onset cirrhosis and neutropenia. Functional analyses supported the variant’s pathogenicity, and sustained clinical benefit after liver transplantation supports its use as a potential therapeutic option for severe FOCAD-related disease. Beyond this rare condition, the study underscores the growing importance of integrating genomic identification, functional validation, and detailed phenotyping in otherwise unexplained liver disease.

Using Global Burden of Disease 2021 data, Yang et al. examined changes in cirrhosis patterns among children, adolescents, and young adults from 2000 to 2021. Although incidence declined in younger age groups, it increased among those aged 15–19 and 20–24 years. The etiological distribution also shifted, with hepatitis B-attributable disease declining and MASLD’s contribution rising among adolescents and young adults. These observations illustrate how the evolving global epidemiology of liver disease may reshape the burden of cirrhosis from an early age.

## 7 Liver decompensation and acute-on-chronic liver failure

The transition from compensated to decompensated cirrhosis represents a major turning point in the natural history of chronic liver disease (22). The development of ascites, variceal bleeding, or hepatic encephalopathy reflects complex interactions among portal hypertension, systemic inflammation, circulatory dysfunction, and declining hepatic reserve (23). In a subset of patients, acute precipitating events may further destabilize this balance, leading to acute-on-chronic liver failure (ACLF), a syndrome characterized by severe systemic inflammation, immune dysfunction, organ failure, and high short-term mortality (24).

Langer et al. investigated circulating extracellular vesicles (EVs) in compensated cirrhosis, acute decompensation, and ACLF, providing mechanistic insight into the immune dysfunction that accompanies advanced liver disease. EVs from patients with cirrhosis showed alterations in surface-marker expression and molecular cargo, with additional changes in ACLF, including enrichment of long non-coding RNAs. Functionally, patient-derived EVs altered T-cell subsets and impaired CD3-positive T-cell viability through mitochondrial dysfunction. These observations identify EV-mediated intercellular communication as a potential contributor to the progressive immune dysfunction that accompanies the transition from compensated cirrhosis to acute decompensation and ACLF.

## 8 Hepatocellular carcinoma

HCC is another major consequence of chronic liver disease, with cirrhosis remaining the principal predisposing condition (25). Prognosis and treatment depend on tumor burden, liver function, portal hypertension, and patient factors, while immune-based therapies increasingly link tumor biology to the immune environment in chronic liver disease (26).

Pantzios et al. addressed the need for practical prognostic tools for patients with unresectable HCC receiving first-line immunotherapy. In a cohort of 100 patients, several baseline characteristics were associated with overall survival (OS), including albumin-bilirubin (ALBI) grade, Barcelona Clinic Liver Cancer (BCLC) stage, macrovascular invasion, up-to-7 criteria, and lymphocyte infiltration. The investigators incorporated the strongest independent prognostic parameters into the ALIVE-IO score, designed to estimate 1-year OS, and internally validated it using receiver operating characteristic (ROC) analysis and 10-fold cross-validation. Patients could then be stratified into distinct risk groups with differences in treatment response and survival. Although external validation remains necessary, this study provides a clinically oriented framework for integrating tumor burden, liver function, and host immune characteristics into prognostication.

## 9 The gut–liver axis

The contributions to this Research Topic also extend beyond the conventional continuum from chronic liver injury to cirrhosis and its complications. Increasing attention is being directed toward the interactions among the intestinal microbiome, intestinal barrier, immune system, and liver. Disruption of this gut–liver axis may influence hepatic inflammation and immune responses across multiple liver diseases and contribute to infectious complications, adding another dimension to the complex interplay between hepatic and extrahepatic systems (27).

Bai et al. examined the emerging role of the gut–liver axis in invasive liver abscess. Integrating microbiomic, transcriptomic, proteomic, and metabolomic evidence, the authors describe how intestinal barrier dysfunction and microbial dysbiosis may facilitate bacterial translocation and alter bile acid and short-chain fatty acid metabolism. These processes may promote hepatic immune dysregulation and inflammation and interact with bacterial virulence mechanisms. Their proposed bacterium–inflammation–virulence framework illustrates how host–microbial interactions may contribute to liver disease and highlights the microbiota and intestinal barrier as potential therapeutic targets.

## 10 Conclusion

Taken together, this Research Topic demonstrates that immune dysregulation links disease initiation, progression, and therapeutic response across viral, autoimmune, cholestatic, metabolic, and alcohol-associated liver diseases. Despite distinct etiologies, viral hepatitis, autoimmune and cholestatic disorders, MASLD, and ALD converge on inflammation, immune dysregulation, oxidative stress, cellular injury, and fibrogenesis. Persistent injury can lead to cirrhosis, portal hypertension, decompensation, ACLF, and hepatocarcinogenesis.

Several contributions go beyond mechanistic descriptions to identify candidate biomarkers, therapeutic targets, and approaches to treatment personalization. Integrating molecular and immunological insights with clinical phenotyping will be essential to translating these advances into improved outcomes.
